# Roles of lactate and base deficit in predicting traumatic coagulopathy

**DOI:** 10.1371/journal.pone.0327321

**Published:** 2025-07-11

**Authors:** Wook Tae Yang, Il Jae Wang, Suck Ju Cho, Seok-Ran Yeom, Sung-Wook Park, Won Ung Tae, Tae Sik Goh, Up Huh, Dongman Ryu, Chanhee Song, Young Mo Cho

**Affiliations:** 1 Department of Emergency Medicine, Pusan National University School of Medicine and Biomedical Research Institute, Pusan National University Hospital, Busan, Republic of Korea; 2 Department of Orthopedic Surgery, Pusan National University School of Medicine and Biomedical Research Institute, Pusan National University Hospital, Busan, Republic of Korea; 3 Department of Thoracic and Cardiovascular Surgery, Pusan National University School of Medicine and Biomedical Research Institute, Pusan National University Hospital, Busan, Republic of Korea; 4 Medical Research Institute, Pusan National University, Busan, Republic of Korea; Azienda Ospedaliero Universitaria Careggi, ITALY

## Abstract

Timely and accurate initial assessment of trauma patients can significantly affect future outcomes. This study aimed to compare the predictive value of the lactate level and base deficit for traumatic coagulopathy, in-hospital mortality, and massive transfusion. This retrospective, observational, single-center study included patients who visited a trauma center from 2016 to 2020. The primary outcome was traumatic coagulopathy, and the secondary outcomes were in-hospital mortality and massive transfusion. Logistic regression analysis was performed to determine whether the lactate level and base deficit were independent risk factors. The area under the receiver operating characteristic curve was calculated to assess the predictive value of lactate and base deficit. In total, 4,379 patients were included in the study. In the logistic regression analysis, base deficit was identified as an independent risk factor for traumatic coagulopathy, whereas lactate was not. Regarding in-hospital mortality, the lactate level was an independent risk factor, whereas base deficit was not. The area under the curve values for predicting traumatic coagulopathy using lactate levels and base deficit were 0.710 (95% confidence interval [CI], 0.696–0.723) and 0.756 (95% CI, 0.743–0.769), respectively; this difference was statistically significant (p < 0.0001; 95% CI, 0.030–0.0622). Base deficit excelled in traumatic coagulopathy prediction, whereas lactate levels prevailed in mortality prediction. Both markers warrant careful observation in the assessment and management of patients with trauma.

## Introduction

Severe trauma is a widely recognized critical issue on both individual and societal scales, predominantly affecting younger individuals engaged in various societal activities [1,2]. Its consequences include severe injuries, disabilities, and life-threatening situations, which impact education, the economy, and the labor market. Addressing severe trauma at the national level requires substantial investments in emergency medical care and rehabilitation infrastructure.

Timely and accurate initial assessment of trauma patients can significantly affect future outcomes [3]. Traditionally, prognosis predictions have relied on clinical expertise, subjective assessments, or normalizing vital signs during resuscitation. Recent medical advancements have introduced various laboratory markers for predicting prognosis. The lactate level and base deficit (BD) are widely utilized indicators in predicting the prognosis and severity of trauma. Lactate levels provide a direct assessment of anaerobic metabolic byproducts during inadequate blood flow, reflecting intracellular lactic acidosis [4]. BD indicates the balance of acids and alkalis affecting blood pH; it is calculated using pH, pCO_2_, and hemoglobin levels. Although both markers have shown promise in trauma assessment, their comparative predictive values remain inconclusive, with conflicting results reported in the literature [5].

Traumatic coagulopathy (TC) is critically significant in severe trauma cases, affecting an estimated 25–35% of trauma patients [6]. Early and accurate prediction and management are pivotal owing to the associated risks of severe bleeding, multiorgan failure, and increased mortality rates [7]. However, limited research exists on the assessment of lactate levels and BD for predicting TC in patients with trauma.

This study aimed to assess the predictive value of lactate levels and BD for TC. Moreover, we compared the predictive values for mortality and massive transfusion (MT), which showed contradicting results in previous studies. We hypothesized that: a) both lactate and BD are useful indicators for predicting TC; and b) lactate, which is a direct measure from bodily fluids and is less influenced by other factors, may outperform BD in predicting TC, in-hospital mortality, and MT.

## Materials and methods

### Study design and setting

This retrospective, single-center study evaluated the prognostic power of lactate levels and BD in patients with trauma. The study was conducted at the 1,400-bed trauma center at our hospital. This center is one of the largest trauma centers in Korea and admits approximately 1,000 patients with an Injury Severity Score (ISS) of ≥15 annually. This study was approved by the Institutional Review Board of our hospital (approval no. 2312-001-133). Medical data was accessed from December 5, 2023 to April 30, 2024 for this study. The requirement for informed consent was waived because patient information was anonymized, and the study was retrospective in nature.

### Study population

This study included patients who presented to the trauma center between 2016 and 2020. Patients aged <16 years, those with missing data, and those with prehospital cardiac arrest were excluded.

### Data and outcome variables

Data for this study were obtained from the Korean Trauma Data Bank and the electronic medical records of the trauma center. The data collected included age, sex, injury mechanism, Glasgow Coma Scale score, systolic blood pressure, transfusion requirement, MT, ISS, Revised Trauma Score (RTS), and in-hospital mortality. The following laboratory data were also collected: prothrombin time-international normalized ratio (PT-INR), activated partial thromboplastin time (aPTT), lactic acid levels, and BD. PT-INR, Lactate acid levels and BD were obtained using point of care testing; PT-INR and lactate was measured using the CA-1500 analyzer (SYSMEX) and GEM-5000 device (WERFEN), respectively, and BD was analyzed with the c702 analyzer (ROCHE). The primary outcome was TC, which was defined as a PT-INR of ≥1.2. The secondary outcomes were in-hospital mortality and MT [8–14]. MT was defined as the transfusion of 10 or more units of packed red blood cells (PRBCs) within 24-hour [15].

### Statistical analysis

Categorical variables are reported as frequencies and percentages, and continuous variables are described as medians (interquartile ranges; IQR) or means (standard deviations; SD), depending on the normality of the distribution. We performed the Shapiro-Wilk test for normal distribution. Categorical variables were compared using the t-test, and continuous variables were compared using the Mann–Whitney U test. Patients were divided into two groups according to the presence or absence of TC. Logistic regression analysis was performed to assess whether lactate levels and BD were independent risk factors for outcomes. A receiver operating characteristic (ROC) curve was drawn, and the area under the ROC curve (AUROC) was calculated to evaluate the predictive abilities of lactate and BD. Delong’s test was used for ROC curves comparison [16,17]. We conducted a sample size analysis for the ROC with the following parameters: a desired AUC of 0.75 (indicating moderate accuracy), a significance level (α) of 0.05, and a power (1-β) of 0.20 for a two-tailed test. About traumatic coagulopathy, we set the ratio of the negative to positive group at 3 based on previous research. Then we calculated the required sample size to be approximately 56 patients [18–20]. Statistical analyses were performed using R 4.1.3 (R Foundation for Statistical Computing, Vienna, Austria).

## Results

### Patient characteristics

**Fig 1:**
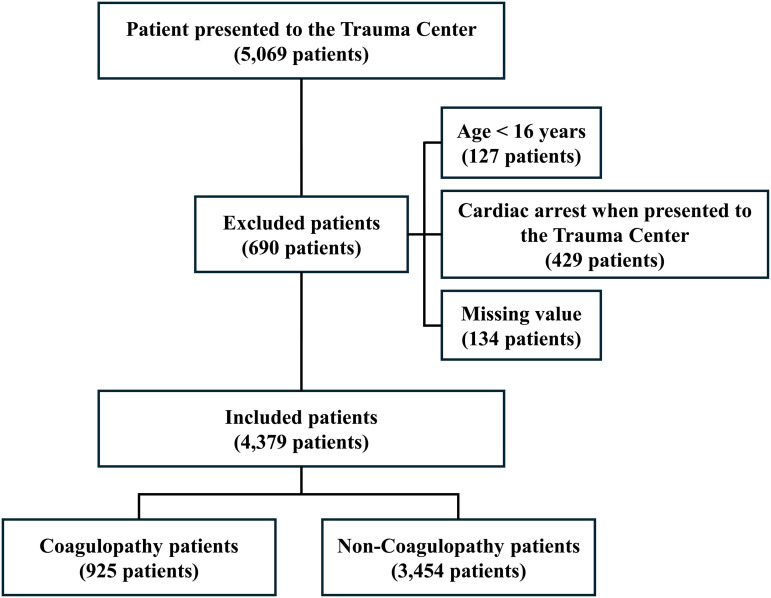
Flow chart of patient selection.

In total, 5,069 patients presented to the trauma center from 2017 to 2020. Among these, 690 patients were excluded based on the following exclusion criteria: (1) age < 16 years, (2) cardiac arrest when presented to the trauma center, and (3) missing values. Finally, 4,379 patients were included in this study. The mean age was 55.78 (17.85) years; there were 3,346 male (76.4%) and 1,033 female (24.6%) patients. The most common type of injury mechanism was traffic accidents (2,183 patients; 49.85%), followed by falls from a height (27.06%), blunt trauma by an object (7.65%), penetration (6.87%), ground-level fall (5.60%), and other causes (2.97%). In total, 359 patients (8.2%) underwent MT. The mean ISS was 19.63 (11.61), and median (IQR) was 18.00 (11.00, 26.00), and the mean RTS was 8.49 (3.98). The in-hospital mortality rate was 10% (437 patients). The mean PT-INR, aPTT, lactate levels, and BD were 1.15 (0.48), 28.65 (11.71) s, 3.36 (2.76), and 1.59 (5.41), respectively. Patient characteristics are summarized in Fig 1 and Table 1, and the characteristics of enrolled patients according to MT and in-hospital mortality status are detailed in S1 and S2 Tables.

**Table 1:** Characteristics of enrolled patients according to coagulopathy status.

Variable	Total (n = 4,379)	Coagulopathy (n = 925)	Non-coagulopathy (n = 3,454)	p-value
Age (y), Mean (SD)	55.78 (17.85)	57.47 (19.83)	55.32 (17.25)	<0.0001
Sex, n (%)				0.04
Male	3,346 (76.4)	683 (73.8)	2,663 (77.1)	
Female,	1033 (23.6)	242 (26.2)	791 (22.9)	
Injury mechanism, n (%)				<0.0001
Traffic accident	2,183 (49.85)	471 (50.92)	1,712 (49.57)	
Fall from height	1,185 (27.06)	285 (30.81)	900 (26.06)	
Ground level fall	245 (5.60)	39 (4.22)	206 (5.96)	
Blunt trauma by object	335 (7.65)	41 (4.43)	294 (8.51)	
Penetrating	301 (6.87)	43 (4.65)	258 (7.47)	
Etc	130 (2.97)	46 (4.97)	84 (2.43)	
Hospital GCS, Mean (SD)	12.64 (3.94)	10.59 (4.91)	13.19 (3.43)	<0.0001
Hospital systolic blood pressure, Mean (SD)	116.99 (35.31)	95.77 (39.47)	122.67 (31.80)	<0.0001
Transfusion, n (%)	2,014 (46)	738 (79.8)	1,276 (36.9)	<0.001
Massive Transfusion, n (%)	359 (8.2)	246 (26.9)	110 (3.2)	<0.0001
ISS, Mean (SD)	19.63 (11.61)	26.92 (12.72)	17.68 (10.47)	<0.0001
RTS, Mean (SD)	8.49 (3.98)	7.76 (4.59)	8.68 (3.78)	<0.0001
In-hospital mortality, n (%)	437 (10)	257 (27.8)	180 (5.2)	<0.0001
PT INR, Mean (SD)	1.15 (0.48)	1.55 (0.93)	1.05 (0.07)	<0.0001
aPTT, Mean (SD)	28.65 (11.71)	39.33 (21.46)	25.84 (3.77)	<0.0001
Lactate, Mean (SD)	3.36 (2.76)	5.28 (3.89)	2.84 (2.09)	<0.0001
Base deficit, Mean (SD)	1.59 (5.41)	5.88 (6.75)	0.44 (4.32)	<0.0001

SD: Standard deviation; GCS: Glasgow coma scale; ISS: Injury severity score; RTS: Revised trauma score; PT INR: Prothrombin time international normalized ratio; aPTT: Activated partial thromboplastin time.

### Comparison of TC and non-TC

We compared two groups: TC and non-TC. Patients in the TC group were older and more likely to be male than those in the non-TC group. The TC group received more MT (26.9%) than the non-TC group (3.2%). The mean ISS of the TC and non-TC groups were 26.92 and 17.68, respectively. The in-hospital mortality rate was higher in the TC group (27.8%) than that in the non-TC group (5.2%). The means of the lactate levels and BD were 5.28 (3.89) and 5.88 (6.75) in the TC group and 2.84 (2.09) and 0.44 (4.32) in the non-TC group, respectively (considerably higher in the TC group).

### Logistic regression analysis

We performed logistic regression analysis after adjusting for age, sex, and ISS, and injury mechanism to evaluate whether lactate and BD were independent risk factors for TC, in-hospital mortality, and MT. Table 2 shows the results of the logistic regression analysis to identify the independent effects of lactate level and BD on outcomes. For TC, BD was an independent risk factor (adjusted odds ratio [OR], 1.17; 95% confidence interval [CI], 1.14–1.20; p < 0.0001). For in-hospital mortality, lactate was an independent risk factor (adjusted OR, 1.21; 95% CI, 1.14–1.27; p < 0.0001). For MT, both lactate (adjusted OR, 1.16; 95% CI, 1.10–1.24; p < 0.0001) and BD (adjusted OR, 1.09; 95% CI, 1.05–1.12; p < 0.0001) were independent risk factors.

**Table 2:** Logistic regression to predict traumatic coagulopathy, in-hospital mortality, and massive transfusion.

Variable	Odds ratio	95% CI	p-value
**Traumatic coagulopathy**			**0.5618**
Age	1.01	1.01–1.02	<0.0001
Sex	1.10	0.91–1.32	0.40
Injury mechanism			
Traffic accident	0.75	0.48–1.15	0.18
Fall from height	0.85	0.54–1.32	0.46
Ground level fall	0.73	0.42–1.28	0.27
Blunt trauma by object	0.45	0.26–0.79	0.01
Penetrating	0.63	0.36–1.11	0.11
ISS	1.05	1.05–1.06	<0.0001
Lactate	1.03	0.98–1.08	0.21
Base deficit	1.17	1.14–1.20	<0.0001
**In-hospital mortality**			**0.0303**
Age	1.04	1.03–1.04	<0.0001
Sex	1.01	0.78–1.30	0.94
Injury mechanism			
Traffic accident	0.39	0.24–0.61	<0.0001
Fall from height	0.36	0.22–0.58	<0.0001
Ground level fall	0.37	0.19–0.70	0.00
Blunt trauma by object	0.25	0.13–0.50	0.00
Penetrating	0.08	0.03–0.25	<0.0001
ISS	1.07	1.06–1.08	<0.0001
Lactate	1.21	1.15–1.27	<0.0001
Base deficit	1.02	0.98–1.04	0.30
**Massive transfusion**			**0.0015**
Age	1.01	1.00–1.02	0.02
Sex	1.25	0.94–1.66	0.12
Injury mechanism			
Traffic accident	5.33	2.11–13.43	0.00
Fall from height	3.75	1.47–9.57	0.01
Ground level fall	0.87	0.20–3.85	0.86
Blunt trauma by object	4.65	1.64–13.17	0.00
Penetrating	3.52	1.17–10.56	0.03
ISS	1.07	1.06–1.08	<0.0001
Lactate	1.16	1.10–1.24	<0.0001
Base deficit	1.09	1.05–1.12	<0.0001

CI: Confidence interval; ISS: Injury severity score.

### ROC analysis

#### Traumatic coagulopathy.

**Fig 2:**
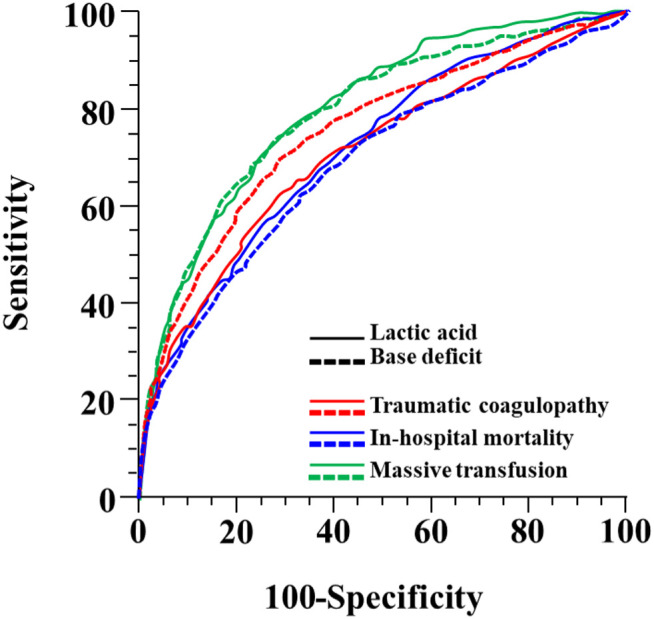
Receiver operating curve (ROC) of lactate levels and base deficit to predict traumatic coagulopathy, in-hospital mortality and massive transfusion.

ROC analysis was performed, and AUROCs were calculated to assess the predictive values of lactate and BD for TC (Fig 2). The AUROCs for predicting TC using lactate levels and BD were 0.710 (95% CI, 0.696–0.723) and 0.756 (95% CI, 0.743–0.769), respectively. BD showed a significantly higher AUROC than lactate (p < 0.0001; 95% CI, 0.030–0.0622).

#### In-hospital mortality and massive transfusion.

ROC analysis was performed to predict in-hospital mortality and MT, and AUROCs were calculated (Fig 2). The AUROCs for predicting in-hospital mortality using lactate levels and BD were 0.716 (95% CI, 0.703–0.730) and 0.688 (95% CI, 0.674–0.701), respectively. Lactate levels showed a significantly higher AUROC than BD (p = 0.0102; 95% CI, 0.0068–0.0509). The AUROC of lactate for predicting MT was 0.801 (95% CI, 0.778–0.824), which was higher than that (0.793 [95% CI, 0.767–0.818]) of BD; however, the difference was not statistically significant (p = 0.3497; 95% CI, −0.0092 to 0.0259).

## Discussion

In this study, we compared the predictive values of lactate levels and BD as indicators of TC, mortality, and MT following severe trauma. The key findings of this study were as follows:

(a) The prevalence of MT in patients with trauma was 21.1%.
(b) BD was identified as an independent risk factor for predicting TC and demonstrated statistically superior predictive ability compared to lactate. However, the absolute difference in AUROC values between BD and lactate was not substantial.
(c) For predicting mortality, lactate levels were an independent risk factor with superior predictive ability to BD.
(d) Both lactate levels and BD demonstrated strong predictive values for predicting MT in trauma patients.

Lactate level and BD have proven to be valuable prognostic tools for patients with trauma [21–25]. Moreover, the applicability of these tools in point-of-care testing allows rapid results, which are crucial for the management of patients with trauma. Lactate is a byproduct of anaerobic metabolism that occurs after glycolysis, in which pyruvate is converted into lactic acid through the enzymatic action of lactate dehydrogenase. In the aftermath of trauma, factors such as tissue damage, hemorrhage, and diminished oxygen supply due to insufficient perfusion can result in increased lactate production [26]. Catecholamines, such as adrenaline and noradrenaline, play a significant role in regulating lactate production. They stimulate glycogenolysis and glycolysis, increasing the production of pyruvate, which is converted to lactate under anaerobic conditions. BD serves as an indicator of insufficient levels of alkaline substances in the bloodstream. This signifies the quantity of alkali needed to maintain 1 L of arterial blood at a pH of 7.4, making it an indispensable parameter for evaluating blood pH and acid-base equilibrium [27].

Lactate and BD have been reported as prognostic factors in patients with trauma, predicting outcomes such as admission to the intensive care unit, transfusion requirements, and mortality [22,28,29]. Several studies have shown that BD is superior to lactate in terms of predicting outcomes. In a study by Davis et al., only BD was associated with transfusion [30]. In a subsequent study, Davis et al. demonstrated that both BD and lactate levels were associated with shock, high ISS, prolonged intensive care unit admission, and increased transfusion requirements. BD is a more accurate predictor of mortality than lactate level [31]. The results of these studies are inconsistent with ours. In contrast, Mikulascheks et al. suggested that lactate is a better predictor of resuscitation than BD in patients with trauma [32]. Gale et al. demonstrated that early lactate levels were a superior predictor of in-hospital mortality to BD [33]. Similarly, Qi et al. found that lactate was a superior predictor of mortality to BD in trauma patients [34]. This hypothesis is supported by our findings. In our study, lactate levels were superior to BD in predicting mortality.

In the present study, both lactate levels and BD were effective in predicting MT, which is consistent with previous findings. Takehiro et al. conducted a study to assess the predictive value of BD for MT. They compared the predicted value of BD with the Agitated Behavior Scale, trauma-associated severe hemorrhage, and fibrinogen scores. Their results showed BD as a strong independent predictor for MT [35]. Moreover, the study conducted by Magdalene et al., which involved 3,468 patients with trauma, demonstrated a strong correlation between lactate and MT [36].

TC occurs in more than one-third of patients with severe trauma, and it is associated with a very poor prognosis [37]. The incidence of TC involves various mechanisms such as consumptive coagulopathy, excessive fibrinolysis, and activation of inflammatory pathways [38,39]. Early recognition and intervention are crucial in the management of TC. To the best of our knowledge, only one study has compared lactate levels and BD in predicting TC. In 2013, Cheddie et al. conducted a study that included 28 patients and compared lactate levels and BD as predictors of TC. They found that BD was a superior indicator to lactate for predicting TC [26]. However, logistic regression and ROC analyses were not performed. In our study, we included a substantial cohort of 4,379 patients and performed logistic regression and ROC analyses.

We hypothesized that the lactate level is a better predictor for TC than BD. The reason for this prediction is that lactate is less influenced by external factors than BD [40]. BD can be influenced by the administration of crystalloid solutions or blood transfusions, as preservatives and blood components may alter the acid-base balance. Additionally, mechanical ventilation or oxygen supplementation can affect CO2 levels in the body, leading to variations in BD values. These factors should be considered when interpreting BD as a clinical marker. In our study, although BD demonstrated statistically superior predictive ability compared to lactate, the absolute difference in AUROC values (0.76 vs. 0.71) is not large enough to definitively conclude that BD is clinically superior to lactate in predicting TC. This suggests that both markers should be considered collectively when evaluating patients. Furthermore, multicenter studies are warranted to validate these findings and enhance their reliability across diverse clinical settings.

This study had some limitations. First, this was a retrospective study, and the possibility of selection bias cannot be ruled out. Second, this study was conducted at a single center, raising uncertainty regarding the generalizability of the results owing to regional variations. Finally, lactate levels and BD may be beneficial for continuous measurements; however, in our study, we used only the initial values. Finally, there is no information on anticoagulant use [41]. Future research should consider prospective, multi-center investigations to mitigate selection bias and enhance generalizability. Additionally, exploring regional variability, implementing continuous monitoring, conducting subgroup analyses, and integrating additional variables could refine predictive models and contribute to a more nuanced understanding of lactate level and BD in traumatic contexts.

## Conclusions

BD proved superior in predicting TC to lactate; however, lactate emerged as a superior predictor for mortality. In predicting MT, both BD and lactate demonstrated strong predictive capabilities. Therefore, careful monitoring of both markers is crucial for evaluating and managing patients with trauma.

## Supporting information

S1 and S2 Table Characteristics of enrolled patients according to massive transfusion and in-hospital mortality status. (DOCX)
