# Supplementary material for: Roles of lactate and base deficit in predicting traumatic coagulopathy
Source: PLoS One. 2025 Jul 11;20(7):e0327321. doi: 10.1371/journal.pone.0327321 (PMC12250580; doi:10.1371/journal.pone.0327321)
Supplement: S1 and S2 Table — (DOCX) [file pone.0327321.s001.docx]

**S1 Table.** Characteristics of enrolled patients according to massive transfusion status

| Variables | Total(n=4,379) | Massive Transfusion (n=359) | Non- Massive Transfusion (n=4020) | P-value |
| --- | --- | --- | --- | --- |
| Age,  Mean(SD)  Median(IQR) | 55.78(17.85) 58.00(44.00,68.00) | 56.21 (18.34)  59.00(46.00,69.00) | 55.73 (17.80)  58.00(44.00,68.00) | <0.0001 |
| Sex, n (%)  Male  Female | 3346(76.41)  1033(23.59) | 255 (71.03)  104 (28.97) | 3091 (76.89)  929 (23.11) | 0.01 |
| Injury mechanism, n (%)  Traffic accident  Fall from height  Ground level fall  Blunt trauma by object  Penetrating  Etc | 2183(49.85)  1185(27.06)  245(5.60)  335(7.65)  301(6.87)  130(2.97) | 215(59.89)  99(27.58)  3(0.83)  22(6.13)  13(3.62)  7(1.95) | 1968(48.96)  1086(27.01)  242(6.02)  313(7.79)  288(7.16)  123(3.06) | <0.0001 |
| Hospital GCS,  Mean(SD)  Median(IQR) | 12.64(3.94)  15.00(12.00, 15.00) | 9.12(4.94)  8.00(4.00, 15.00) | 12.95(3.67)  15.00(13.00, 15.00) | <0.0001 |
| Hospital systolic blood pressure,  Means(SD)  Median  (IQR) | 116.99(35.31)  120.00  (100.00,140.00) | 79.34(39.62)  70.00  (50.00,100.00) | 120.35(32.87)  120.00  (100.00,140.00) | <0.0001 |
| Transfusion, n (%)  NO  YES | 2365(54.01)  2014(45.99) | 0(0)  359(100) | 2365(58.83)  1655(41.17) |  |
| Coagulopathy, n (%)  NO  YES | 3454(78.88)  925(21.12) | 110(30.64)  249(69.36) | 3344(83.18)  676(16.82) |  |
| ISS,  Mean(SD)  Median(IQR) | 19.63(11.61)  18.00 (11.00,26.00) | 32.35(0.63)  33.00(24.00,38.00) | 18.51(0.17)  17.00(10.00,25.00) | <0.0001 |
| RTS,  Mean(SD)  Median(IQR) | 8.49(3.98)  10.00(4.00,12.00) | 6.12(4.34)  8.00(0.00,1.00) | 8.70(3.88)  11.00 (4.00,12.00) | <0.0001 |
| In-hospital mortality, n (%)  NO  YES | 3942(90.02)  437(9.98) | 199(55.43)  160(44.57) | 3743(93.11)  277(6.89) |  |
| PT_INR,  Mean(SD)  Median(IQR) | 1.15(0.48)  1.07(1.01,1.17) | 1.63(1.21)  1.31(1.17,1.63) | 1.11(0.31)  1.06(1.00,1.15) | <0.0001 |
| aPTT,  Mean(SD)  Median(IQR) | 28.65(11.71)  26.00(23.60,29.80) | 43.47(1.54)  32.70(36.80,46.80) | 27.36(7.57)  25.80(23.50,29.20) | <0.0001 |
| Lactate acid,  Mean(SD)  Median(IQR) | 3.36(2.76)  2.60(1.60,4.10) | 6.60(3.98)  5.50(3.40,9.20) | 3.07(2.42)  2.40(1.50,3.80) | <0.0001 |
| Base deficit,  Mean(SD)  Median(IQR) | 1.59(5.41)  0.50(-1.19,4.10) | 7.80(7.07)  6.90(2.50,12.08) | 1.04(4.87)  0.30(-2.10,3.30) | <0.0001 |

SD: Standard deviation; GCS: Glasgow coma scale; ISS: Injury severity score; RTS: Revised trauma score; PT INR: Prothrombin time international normalized ratio; aPTT: Activated partial thromboplastin time

**S2 Table.** Characteristics of enrolled patients according to in-hospital mortality status

| Variables | Total  (n=4,379) | In-hospital mortality  (n=437) | survival  (n=3942) | P-value |
| --- | --- | --- | --- | --- |
| Age,  Mean(SD)  Median(IQR) | 55.78 (17.85) 58.00(44.00,68.00) | 63.31(17.03)  66.00(55.00,77.00) | 54.94(17.74)  58.00(43.00,68.00) | <0.0001 |
| Sex, n (%)  Male  Female | 3346(76.41)  1033(23.59) | 320(73.23)  117(26.77) | 3026(76.76)  916(23.24) | 0.10 |
| Injury mechanism, n (%)  Traffic accident  Fall from height  Ground level fall  Blunt trauma by object  Penetrating  Etc | 2183(49.85)  1185(27.06)  245(5.60)  335(7.65)  301(6.87)  130(2.97) | 232(53.09)  123(28.14)  21(4.81)  19(4.35)  4(0.91)  38(8.70) | 1951(49.49)  1062(26.94)  224(5.68)  316(8.02)  297(7.54)  92(2.33) | <0.0001 |
| Hospital GCS,  Mean(SD)  Median(IQR) | 12.64(3.94)  15.00(12.00, 15.00) | 6.84(4.35)  5.00(3.00,10.00) | 13.28(3.31)  15.00(14.00,15.00) | <0.0001 |
| Hospital systolic blood pressure,  Means(SD)  Median  (IQR) | 116.99(35.31)  120.00  (100.00,140.00) | 108.14(52.38)  100.00  (70.00,142.50) | 117.97(32.74)  120.00  (100.00,140.00) | <0.0001 |
| Transfusion, n (%)  NO  YES | 2365(54.01)  2014(45.99) | 78(17.85)  359(82.15) | 2287(58.02)  1655(41.98) |  |
| Coagulopathy, n (%)  NO  YES | 3454(78.88)  925(21.12) | 180(41.19)  257(58.81) | 3274(83.05)  668(16.95) |  |
| ISS,  Mean(SD)  Median(IQR) | 19.63(11.61)  18.00(11.00,26.00) | 30.48(11.93)  29.00(25.00,38.00) | 18.43(10.93)  17.00(10.00,25.00) | <0.0001 |
| RTS,  Mean(SD)  Median(IQR) | 8.49(3.98)  10.00(4.00,12.00) | 5.93(4.53)  8.00(0.00,10.00) | 8.77(3.82)  11.00(4.00,12.00) | <0.0001 |
| Massive Transfusion, n (%)  NO  YES | 4020(91.80)  359(8.20) | 277(63.39)  160(36.61) | 3743(94.95)  199(5.05) |  |
| PT_INR,  Mean(SD)  Median(IQR) | 1.15(0.48)  1.07(1.01,1.17) | 1.61(1.32)  1.26(1.10,1.63) | 1.10(0.19)  1.06(1.00,1.15) | <0.0001 |
| aPTT,  Mean(SD)  Median(IQR) | 28.65(11.71)  26.00(23.60,29.80) | 42.73(1.29)  33.50(27.50,45.50) | 27.14(7.35)  25.70(23.50,29.00) | <0.0001 |
| Lactate acid,  Mean(SD)  Median(IQR) | 3.36(2.76)  2.60(1.60,4.10) | 5.72(4.11)  4.20(2.60,8.13) | 3.10(2.43)  2.40(1.50,3.80) | <0.0001 |
| Base deficit,  Mean(SD)  median(IQR) | 1.59(5.41)  0.50(-1.19,4.10) | 5.54(7.51)  3.80(0.40,9.83) | 1.15(4.94)  0.40(-2.10,3.70) | <0.0001 |

SD: Standard deviation; GCS: Glasgow coma scale; ISS: Injury severity score; RTS: Revised trauma score; PT INR: Prothrombin time international normalized ratio; aPTT: Activated partial thromboplastin time
